# Supplementary material for: Environmental complexity is more important than mutation in driving the evolution of latent novel traits in E. coli
Source: Nat Commun. 2022 Oct 6;13:5904. doi: 10.1038/s41467-022-33634-w (PMC9537139; doi:10.1038/s41467-022-33634-w)
Supplement: Supplementary file 1 — Supplementary Information [file 41467_2022_33634_MOESM1_ESM.pdf]

Supplementary material

Environmental complexity is more important than mutation in driving the evolution of latent novel traits in *E. coli*

Shraddha Karve[1,5] and Andreas Wagner[1, 2, 3, 4]

Table S1: List of antimicrobials characterizing those phenotyping environments in which the wild-type or the mutator strain is inviable. For inviability, we require that none of the two tested clones shows an OD_{600} exceeding 0.3 after 48h of growth. The wild-type strain was inviable in 95 phenotyping environments while the mutator strain was inviable in 58 phenotyping environments. The 52 environments where both ancestors were inviable are shown in blue.

Wild-type	Mutator
1,10-Phenanthroline	1,10-Phenanthroline
1-chloro-2,4-dinitrobenzene	2,4-Dinitrophenol
1-hydroxy pyridine-2-thione	2-Nitroimidazole
2,2-Dipyridyl	3,4-Dimethoxy-benzyl alcohol
2,4-Dinitrophenol	3,5-Dinitro-benzene
2-Nitroimidazole	4-aminopyridine
3,4-Dimethoxy-benzyl alcohol	4-Hydroxy-coumarin
3,5-Dinitro-benzene	8-Hydroxy-quinoline
4-aminopyridine	Alexidine
4-Hydroxy-coumarin	Amitriptyline
5,7-Dichloro-8-hydroxyquinoline	Atropine
8-Hydroxy-quinoline	Benzethonium chloride
Alexidine	Cadmium chloride
Amitriptyline	Chloramphenicol
Antimony(III)chloride	Chlorpromazine
Atropine	Cinnamic acid
Azathioprine	Cobalt chloride
Benzethonium chloride	Coumarin
Blasticidin S	Cupric chloride
Boric acid	D,L-Propranolol
Cadmium chloride	D,L-Thioctic acid
Carbenicillin	Dodecyltrimethyl ammonium bromide
Cefotaxime	Dodine
Cefuroxime	Domiphen bromide
Cetylpyridinium chloride	D-serine
Chlorpromazine	Gallic acid
Cinnamic acid	Guanidine hydrochloride
Cinoxacin	Iodo acetate
Cloxacillin	Josamycin
Ciprofloxacin	Lidocaine
Cobalt chloride	Lithium chloride
Collistin	Minocycline
Coumarin	Niaproof
Cupric chloride	Nickel chloride
D,L-Propranolol	Ornidazole
D,L-Thioctic acid	Orphenadrine

Diamide	Oxycarboxin
Dodecyltrimethyl ammonium bromide	Phenethicillin
Dodine	Phenylarsine oxide
Domiphen bromide	Pridinol
D-serine	Proflavine
Enoxacin	Promethazine
Fusaric acid	Puromycin
Gallic acid	Rolitetracycline
Glycine hydroxamate	Sanguinarine
Harmane	Semicarbazide
Hygromycin B	Sodim metavanadate
Iodo acetate	Sodium azide
Josamycin	Sodium bromate
Ketoprofen	Sodium Caprylate
L-Aspartic-B-hydroxamate	Sodium metasilicate
Lauryl sulfobetaine	Sodium nitrite
Lawsone	Sodium orthovanadate
Lidocaine	Sodium salicylate
Lithium chloride	sodium-m-periodate
Lomefloxacin	Spectinomycin
Minocycline	Tetrazolium violet
Nafcillin	Thiamphenicol
Nalidixic acid	
Nickel chloride	
Norfloxacin	
Novobiocin	
Ofloxacin	
Orphenadrine	
Oxacillin	
Oxycarboxin	
Patulin	
Phenethicillin	
Phenylarsine oxide	
Polymyxin B	
Pridinol	
Promethazine	
Puromycin	
Sanguinarine	
Semicarbazide	
Sodim metavanadate	
Sodium azide	
Sodium bromate	
Sodium Caprylate	
Sodium cyanate	

Sodium metasilicate	
Sodium nitrite	
Sodium orthovanadate	
Sodium salicylate	
Sodium selenite	
Sodium tungstate	
Sodium-m-arsenite	
sodium-m-periodate	
Spectinomycin	
Spiramycin	
Tetrazolium violet	
Thiamphenicol	
Trimethoprim	
Tylosin	
Vancomycin	

Supplementary note S1:

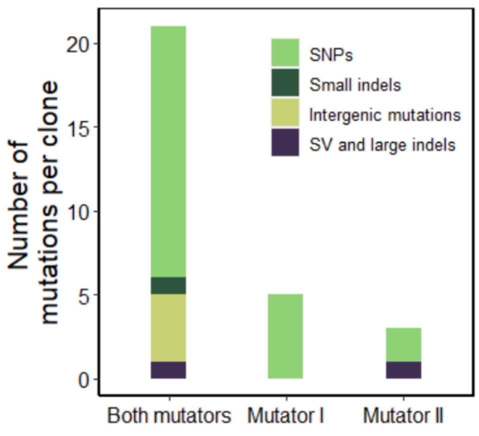

Figure S1: Types of mutations in the two ancestral clones of the mutator strain. We sequenced the genomes of both our ancestral mutator clones and identified DNA variants in their genomes in which they differed from the previously reported sequence of the wild-type strain[1]. We observed 29 such mutations, 21 of which were present in both mutator clones. The first mutator clone harboured five additional mutations, while the second clone harboured three additional mutations. We classified the 29 mutations into four categories. Twenty-two of them were single nucleotide polymorphisms (SNPs), the most common type of mutations. One mutation was a small deletion (2 bp) in the coding region. Two small deletions (smaller than 50bp) and three SNPs occurred in intergenic regions. One mutations was a large insertion of 103bp. Source data are provided as a Source Data file.

We identified those mutations that were absent from the wild-type ancestral strain but present in at least one clone of our ancestral mutator strain. We observed 29 such mutations, 21 of which were present in both mutator clones (Figure S1). One of these 21 mutations is the 103 bp insertion upstream of the gene *mutL* that is responsible for the ten-fold higher mutation rate of the mutator strain [2].

Among the remaining twenty-eight mutations, we aimed to identify candidates to help explain why the mutator is viable in more phenotyping environments than the wild-type strain[1]. We identified five such candidates, four of which were shared by both mutator clones. The first was a single nucleotide change in the gene *hns* that encodes the nucleoid-associated DNA-binding protein H-NS. H-NS plays a major role in the organization of the bacterial chromosome and in the regulation of gene expression [3,4]. H-NS also regulates the expression of the *marA* gene, which encodes a global regulator of multi-drug resistance in *E. coli* [3]. In addition, mutations in *hns* are known to affect drug resistance in *E. coli* [4]. The second mutation was a single nucleotide change in the gene *ldtA*, which encodes L,D-

transpeptidase. β-lactams affect bacterial cell wall synthesis by binding to D,D – transpeptidase, which cross-links the peptidoglycans that form the bacterial cell wall. Production of L,D – transpeptidase can bypass the requirement for D,D – transpeptidase, and confer resistance to a broad spectrum of β-lactam antibiotics [5,6]. The third mutation was a single nucleotide change in *atoS*, which encodes a sensory histidine kinase. Mutations in sensory histidine kinases can confer tolerance to the antibiotic vancomycin, as well as resistance to carbapenem antibiotics and heavy metals such as zinc [7,8]. The fourth shared mutation was a single nucleotide change in the regulatory region of the RNA chaperone Hfq, which regulates the multi-drug efflux pump AcrAB-TolC. Mutations in *hfq* can affect resistance to a wide variety of antimicrobials, such as acriflavine, benzalkonium, cefamandole, chloramphenicol, crystal violet, nalidixic acid, novobiocin, oxacillin and rhodamine [9]. The fifth candidate mutation, which occurred only in one ancestral mutator clone, was a single nucleotide change in the gene *emrY*, which encodes the membrane subunit of a tripartite efflux pump [10,11]. The EmrY/K efflux pump can confer resistance to a wide variety of antibiotics in *E. coli*, including ampicillin, tetracycline, penicillin, erythromycin, and chloramphenicol [10,11]. Overall, this analysis shows that at the beginning of experimental evolution, the mutator strain already harboured several mutations that may have increased its viability in our phenotyping environments.

Table S2: All mutations (column 2 from the left) that we observed in the two mutator clones before the beginning of experimental evolution. The first column on the left indicates whether a mutation occurred in both sequenced clones or in just one of them. Mutations in genes which may affect viability in our phenotyping environment based on available evidence (Supplementary note 1) are marked with ‘ * ’.

Clone	Mutation	Gene	Mutation target
Mutator ancestor I and II	*SNP(T>C)	Downstream of *aspV*	t-RNA of aspartic acid
	*SNP(V235I)	*betA*	Choline dehydrogenase
	SNP(V32A)	*ybdF*	PF04237 family protein
	Small insertion (1bp)	upstream of *serW*	t-RNA of serine
	SNP(T86A)	* *hns*	DNA-binding transcriptional dual regulator
	SNP(L50L)	*uidB*	glucuronide:H(+) symporter
	*SNP(N101D)	* *ldtA*	L,D-transpeptidase
	SNP(V145I)	* *atoS*	sensory histidine kinase
	SNP(P47P)	*yqcC*	DUF446 domain-containing protein
	Small deletion (1bp)	Intergenic region between *ygjR and alx*	*ygjR* codes for putative oxidoreductase and *alx* codes for putative membrane-bound redox modulator
	SNP(P610L)	*fusA*	elongation factor G
	SNP(V226A)	*gntT*	high-affinity gluconate transporter
	Small deletion (2 bp)	*ugpQ*	glycerophosphodiester phosphodiesterase
	SNP(V357V)	*queG*	epoxyqueuosine reductase
	SNP(F172F)	*queG*	epoxyqueuosine reductase
	SNP(T103T)	*amiB*	N-acetylmuramoyl-L-alanine amidase B
	SNP(F236F)	*amiB*	N-acetylmuramoyl-L-alanine amidase B
	Large insertion (103 bp)	Intergenic region between *amiB and mutL*	*amiB* codes for N-acetylmuramoyl-L-alanine amidase B and *mutL* codes for DNA mismatch repair protein MutL
	SNP(C>T)	* Intergenic region between *miaA and hfq*	*miaA* codes for tRNA dimethylallyltransferase and *hfq* codes for RNA-binding protein
	SNP(D94G)	*yjfK*	conserved protein
	SNP(V405A)	*valS*	valine t-RNA ligase
Mutator ancestor I	SNP(L57L)	*yahF*	putative acyl-CoA synthetase
	SNP(T388T)	* *emrY*	tripartite efflux pump membrane subunit
	SNP(T46M)	*relA*	GDP/GTP pyrophosphokinase
	SNP(I64I)	*metH*	cobalamin-dependent methionine synthase
	SNP(S43S)	*malK*	maltose ABC transporter ATP binding subunit
Mutator ancestor II	SNP(S35S)	*secM*	SecA translation regulator
	SNP(A418V)	*zwf*	NADP(+)-dependent glucose-6-phosphate dehydrogenase
	SNP(C>T)	Intergenic region between *nudB and aspS*	*nudB* codes for dihydroneopterin triphosphate diphosphatase and *aspS* codes for aspartate tRNA ligase

Table S3: The table shows the IC_{90} for each antibiotic, the number of days of experimental evolution to which we subjected populations on each antibiotic, and the estimated [12] number of generations for evolution in each antibiotic for populations that had evolved in single antibiotic environments. These number of days and generations derive from a pilot experiment which had shown that bacteria differed in their tolerance to a daily increase in antibiotic dosage, depending on the antibiotic we used. For instance, we could increase the concentration of ampicillin twice as fast as that of trimethoprim without population extinction. This difference among antibiotic environments led to different durations of experimental evolution on different single antibiotics. The previously reported evolution experiments of the wild-type strain[1] shared the starting concentrations, IC_{90} values, and number of days in experimental evolution with our current experiments on the mutator strain.

Antibiotic	Starting concentration in µg/ml	IC_{90} in µg/ml	Number of days	~ Number of generations
Ampicillin	0.25	8	12	108
Azithromycin	0.2	25.6	16	143
Nalidixic acid	0.25	64	19	170
Streptomycin	0.5	16	12	108
Trimethoprim	0.4	409.6	24	215

Table S4: Growth rates per minute (column 3 and 6 from the left) and corresponding doubling times in minutes (column 4 and 7) for the eight evolved mutator populations (column 2) and three clones (column 5) from each evolved population for our five antibiotics (column 1). Growth rates of the two clones that we chose for phenotyping and whole-genome sequencing are marked with '*'.

Antibiotic	Population	Growth rate (per min)	Doubling time (min)	Clone	Growth rate (per min)	Doubling time (min)
Ampicillin	1	0.02308	30	I	0.03073	22.6
				II	*0.01999	34.7
				III	0.02815	24.6
	2	0.01812	38.3	I	0.02472	28
				II	0.01968	35.2
				III	0.02856	24.3
	3	0.02041	34	I	0.01906	36.4
				II	0.02244	30.9
				III	0.02425	28.6
	4	0.02179	31.8	I	0.01923	36
				II	0.01968	35.2
				III	0.02511	27.6
	5	0.01846	37.5	I	*0.02087	33.2
				II	0.02776	25
				III	0.00229	302.8
	6	0.02425	28.6	I	0.01943	35.7
				II	0.02618	26.5
				III	0.02877	24.1
	7	0.02128	32.6	I	0.01815	38.2
				II	0.02177	31.8
				III	0.02307	30
	8	0.02036	34	I	0.01952	35.5
				II	0.01825	38
				III	0.02248	30.8
Azithromycin	1	0.01543	44.9	I	0.02048	33.9
				II	0.01429	48.5
				III	0.00839	82.6
	2	0.01713	40.5	I	0.01507	46
				II	*0.01487	46.6
				III	0.0179	38.7
	3	0.0188	36.9	I	0.01946	35.6
				II	0.02041	34
				III	0.02123	32.7
	4	0.0075	92.4	I	0.00769	90.2
				II	0.00781	88.7
				III	0.00704	98.5
	5	0.01695	40.9	I	0.01772	39.1
				II	0.01595	43.5
				III	0.01825	38
	6	0.01478	46.9	I	*0.01561	44.4
				II	0.01489	46.5
				III	0.01546	44.8
	7	0.01227	56.5	I	0.01439	48.2
				II	0.01983	34.9
				III	0.01387	50
	8	0.01022	67.8	I	0.00989	70.1
				II	0.00751	92.3
				III	0.00807	85.9
Nalidixic acid	1	0.0177	39.2	I	0.02856	24.3
				II	0.02499	27.7
				III	0.02847	24.3
	2	0.02531	27.4	I	*0.01812	38.2
				II	0.02083	33.3
				III	0.01086	63.8
	3	0.02366	29.3	I	*0.01805	38.4
				II	0.02203	31.5
				III	0.01973	35.1
	4	0.01955	35.5	I	0.02095	33.1

				II	0.02047	33.9
				III	0.01876	37
	5	0.02577	26.9	I	0.02144	32.3
				II	0.02676	25.9
				III	0.02144	32.3
	6	0.01563	44.3	I	0.01807	38.4
				II	0.01584	43.7
				III	0.01514	45.8
	7	0.00945	73.3	I	0.01848	37.5
				II	0.01887	36.7
				III	0.01844	37.6
	8	0.01561	44.4	I	0.01788	38.8
				II	0.01578	43.9
				III	0.0166	41.8
Streptomycin	1	0.0176	39.4	I	*0.02057	33.7
				II	0.01919	36.1
				III	0.01587	43.7
	2	0.02231	31.1	I	0.01729	40.1
				II	0.02385	29.1
				III	0.01752	39.6
	3	0.02905	23.9	I	0.02026	34.2
				II	0.01966	35.3
				III	*0.02157	32.1
	4	0.01986	34.9	I	0.01531	45.3
				II	0.01571	44.1
				III	0.01613	43
	5	0.02037	34	I	0.01671	41.5
				II	0.01621	42.7
				III	0.01719	40.3
	6	0.01602	43.3	I	0.01465	47.3
				II	0.01469	47.2
				III	0.01542	45
	7	0.02336	29.7	I	0.01645	42.1
				II	0.01641	42.2
				III	0.01638	42.3
	8	0.02128	32.6	I	0.01524	45.5
				II	0.01528	45.4
				III	0.01418	48.9
Trimethoprim	1	0.02222	31.2	I	0.01722	40.3
				II	0.01283	54
				III	0.01189	58.3
	2	0.01405	49.3	I	0.01819	38.1
				II	0.01824	38
				III	*0.01621	42.8
	3	0.01921	36.1	I	0.01621	42.8
				II	0.01778	39
				III	0.01486	46.6
	4	0.02334	29.7	I	0.01886	36.8
				II	0.02031	34.1
				III	0.01974	35.1
	5	0.02053	33.8	I	0.01602	43.3
				II	0.01772	39.1
				III	0.0171	40.5
	6	0.01727	40.1	I	0.01792	38.7
				II	0.02099	33
				III	0.02367	29.3
	7	0.01927	36	I	*0.01698	40.8
				II	0.01933	35.9
				III	0.01844	37.6
	8	0.01387	50	I	0.01564	44.3
				II	0.01429	48.5
				III	0.01455	47.6

Figure S2:

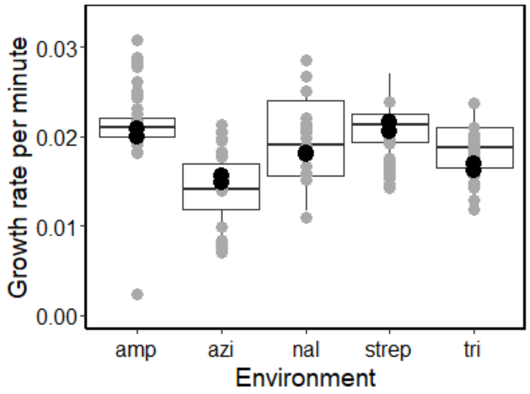

Figure S2: Representative clones for the mutator strain that we evolved in simple evolution environments. Growth rates of eight replicate mutator populations (black boxes, vertical axis) on each of the five antibiotics (horizontal axis) on which they evolved, at the end of experimental evolution. In each box plot, the thick horizontal line represents the mean growth rate of the eight evolved populations, and the lower and upper boundaries of the box represent the first and the third quartile, respectively. Whiskers show 95% confidence intervals. Circles show the growth rates of 24 clones (three randomly chosen clones from each of the eight populations). Black circles represent the growth rates of the two clones, out of 24, that we selected for novel trait assays and for whole-genome sequencing. We estimated all growth rates at the IC_{90} of the respective antibiotic for all populations and clones. Source data are provided as a Source Data file.

Supplementary note S2:

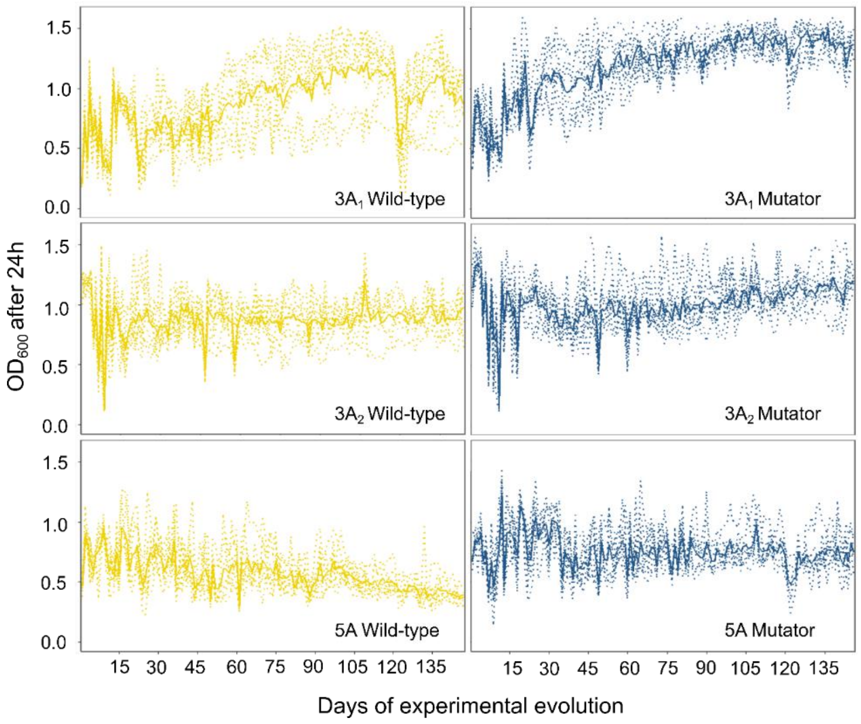

Figure S3: Growth, measured as OD_{600} at the end of 24h, for all the populations evolving in complex environments during ~145 days. We measured OD_{600} (y-axis) every day for ~145 days of experimental evolution (x-axis) for wild-type (yellow) and mutator (blue) populations in environments $3A_1$ (top panels), $3A_2$ (middle panels) and 5A (bottom panels). In every panel, the solid line represents mean OD_{600} for eight replicate populations represented by the dotted lines. Source data are provided as a Source Data file.

We performed experimental evolution in $3A_1$, $3A_2$ and 5A environments in two phases (Methods). The first phase was similar to evolution in simple evolution environments where we transferred 4 µl of culture volume every day, and increased the concentrations of all the antibiotics every second day. We also applied the same criteria used for evolution in simple environments to identify populations with low growth (OD_{600} between 0.2 and 0.3, 20 µl inoculum volume) and extinction (OD_{600} < 0.2, revive 20 µl from the previous day's plate). We observed several extinctions near the end of phase I. Specifically all eight wild-type populations evolving in the $3A_2$ environment went extinct on day 7. Two wild-type populations evolving in the 5A environment went extinct on day 9, while all eight populations went extinct on day 10. One mutator population evolving in the 5A environment went extinct on day 8, while all eight of the mutator populations went extinct in this environment on day 9. We revived these populations from the corresponding previous days' glycerol stocks but the repeated extinctions

demanded a change of strategy for continuing experimental evolution. Overall, phase I lasted 12 days for wild-type and mutator populations evolving in the $3A_1$ environment; 10 days for wild-type populations evolving in the $3A_2$ environment; 13 days for wild-type populations evolving in the 5A environment; and 11 days for mutator populations evolving in the $3A_2$ and 5A environments.

Before beginning the second phase we evolved all wild-type and mutator populations from the end of phase I for four more days on the same antibiotic concentration as on the last day of phase I, in order to avoid extinctions. After these four days, we commenced phase II of experimental evolution, where we increased the concentration of only one antibiotic every day and increased the inoculum volume from the 4 µl of phase I to 100 µl, and from 20 µl to 200 µl for populations with low growth. These changes resulted in reduced selection pressure compared to phase I, and we observed no extinctions during the second phase of experimental evolution. On 7 out of ~135 days of phase II some of the populations showed low growth.

We observed two instances of contamination during this evolution experiment. The first instance occurred on the 33th day of phase II. It affected one of the wild-type populations in the 5A environment, and two mutator populations from the $3A_2$ environment. The second instance affected one of these two mutator populations and occurred on day 37. In each instance we continued the experiment from the last glycerol stock archived before the contamination (Methods).

Phase II lasted 134 days for the wild-type populations evolved in the $3A_2$ and 5A environments, and 135 days for the wild-type populations evolved in the $3A_1$ environments, as well as for mutator populations evolved in the $3A_1$, $3A_2$, and 5A environments.

Table S5: Growth rates per minute (column 3 and 6 from the left) and corresponding doubling times in minutes (column 4 and 7) for the eight evolved wild-type and mutator populations (column 2) and three clones (column 5) from each of the evolved populations for $3A_1$, $3A_2$ and 5A environments (column 1). Growth rates of the two clones that we chose for the novel trait assay, and for whole-genome sequencing are marked with ‘ * ’.

Environment	Population	Growth rate (per min)	Doubling time (min)	Clone	Growth rate (per min)	Doubling time (min)
$3A_1$	Wild-type 1	0.00592	117.1	I	0.00454	152.6
				II	0.00393	176.3
				III	0.00391	177.4
	Wild-type 2	0.0082	84.5	I	*0.00917	75.6
				II	0.00829	83.6
				III	0.00755	91.8
	Wild-type 3	0.01029	67.3	I	0.0073	95
				II	0.01101	63
				III	0.01088	63.7
	Wild-type 4	0.00914	75.8	I	*0.00838	82.7
				II	0.00788	88
				III	0.00953	72.8
	Wild-type 5	0.00755	91.8	I	0.00991	70
				II	0.00657	105.6
				III	0.00584	118.7
	Wild-type 6	0.00883	78.5	I	0.01197	57.9
				II	0.01143	60.7
				III	0.00764	90.8
	Wild-type 7	0.00787	88.1	I	0.0078	88.9
				II	0.00845	82
				III	0.00506	137
	Wild-type 8	0.01037	66.8	I	0.00547	126.7
				II	0.00646	107.2
				III	0.0057	121.7
$3A_1$	Mutator 1	0.01361	50.9	I	0.01056	65.6
				II	0.02124	32.6
				III	*0.01399	49.5
	Mutator 2	0.02898	23.9	I	0.00882	78.6
				II	0.00753	92.1
				III	0.01094	63.3
	Mutator 3	0.01381	50.2	I	0.01342	51.7
				II	*0.00998	69.5
				III	0.01161	59.7
	Mutator 4	0.00709	97.8	I	0.00673	103
				II	0.01254	55.3
				III	0.01016	68.2
	Mutator 5	0.01089	63.6	I	0.0154	45
				II	0.01492	46.4
				III	0.01044	66.4
	Mutator 6	0.01193	58.1	I	0.00552	125.6
				II	0.00866	80
				III	0.01133	61.2
	Mutator 7	0.01286	53.9	I	0.01583	43.8
				II	0.01059	65.5
				III	0.01132	61.2
	Mutator 8	0.00997	69.6	I	0.007	99.1
				II	0.00979	70.8
				III	0.00882	78.5
$3A_2$	Wild-type 1	0.01092	63.5	I	0.01306	53.1
				II	0.01736	39.9
				III	0.01128	61.4
	Wild-type 2	0.01623	42.7	I	0.011	63
				II	0.00825	84
				III	*0.01611	43
	Wild-type 3	0.01571	44.1	I	0.01335	51.9
				II	0.01074	64.5

				III	0.02106	32.9
	Wild-type 4	0.02002	34.6	I	0.01153	60.1
				II	*0.01528	45.4
				III	0.02114	32.8
	Wild-type 5	0.0109	63.6	I	0.01379	50.3
				II	0.01678	41.3
				III	0.02462	28.2
	Wild-type 6	0.0158	43.9	I	0.00776	89.3
				II	0.02387	29
				III	0.02368	29.3
	Wild-type 7	0.0102	68	I	0.00969	71.5
				II	0.0077	90.1
				III	0.01214	57.1
	Wild-type 8	0.0204	34	I	0.01403	49.4
				II	0.01381	50.2
				III	0.0175	39.6
$3A_2$	Mutator 1	0.0138	50.2	I	0.01146	60.5
				II	0.01421	48.8
				III	0.0114	60.8
	Mutator 2	0.01843	37.6	I	0.01957	35.4
				II	0.02341	29.6
				III	0.01882	36.8
	Mutator 3	0.01307	53	I	0.0075	92.4
				II	0.03234	21.4
				III	0.0266	26.1
	Mutator 4	0.01783	38.9	I	0.02821	24.6
				II	0.02532	27.4
				III	*0.01844	37.6
	Mutator 5	0.01779	39	I	0.02552	27.2
				II	0.01713	40.5
				III	0.02485	27.9
	Mutator 6	0.01511	45.9	I	*0.0189	36.7
				II	0.01629	42.5
				III	0.0156	44.4
	Mutator 7	0.02542	27.3	I	0.02875	24.1
				II	0.02061	33.6
				III	0.02596	26.7
	Mutator 8	0.01271	54.5	I	0.01393	49.7
				II	0.00801	86.5
				III	0.02032	34.1
5A	Wild-type 1	0.00588	117.9	I	0.00601	115.4
				II	0.00704	98.5
				III	0.00758	91.4
	Wild-type 2	0.00508	136.6	I	0.00904	76.7
				II	0.00742	93.4
				III	0.00727	95.3
	Wild-type 3	0.00493	140.5	I	0.0056	123.8
				II	0.00668	103.8
				III	0.00451	153.9
	Wild-type 4	0.00679	102.1	I	*0.00568	122
				II	0.00542	127.8
				III	0.00593	116.8
	Wild-type 5	0.00578	120	I	0.00705	98.3
				II	0.00591	117.2
				III	0.00579	119.8
	Wild-type 6	0.00611	113.5	I	0.00665	104.2
				II	0.00689	100.7
				III	*0.00593	116.9
	Wild-type 7	0.00619	111.9	I	0.00695	99.7
				II	0.00639	108.6
				III	0.00607	114.2
	Wild-type 8	0.005	138.7	I	0.00576	120.3
				II	0.00545	127.2
				III	0.0055	126.1
5A	Mutator 1	0.006	115.5	I	0.00613	113.2
				II	0.00696	99.5
				III	0.00559	124.1
	Mutator 2	0.00972	71.3	I	*0.00977	70.9
				II	0.00989	70.1
				III	0.0098	70.7
	Mutator 3	0.01326	52.3	I	0.01783	38.9

				II	0.02076	33.4
				III	0.00718	96.6
	Mutator 4	0.01354	51.2	I	0.01155	60
				II	*0.01406	49.3
				III	0.01437	48.2
	Mutator 5	0.02228	31.1	I	0.01011	68.5
				II	0.01005	68.9
				III	0.00622	111.4
	Mutator 6	0.00753	92.1	I	0.00489	141.7
				II	0.00329	210.6
				III	0.00508	136.5
	Mutator 7	0.00835	83	I	0.00838	82.7
				II	0.00743	93.2
				III	0.00803	86.3
	Mutator 8	0.00882	78.6	I	0.00938	73.9
				II	0.00839	82.6
				III	0.0061	113.6

Figure S4:

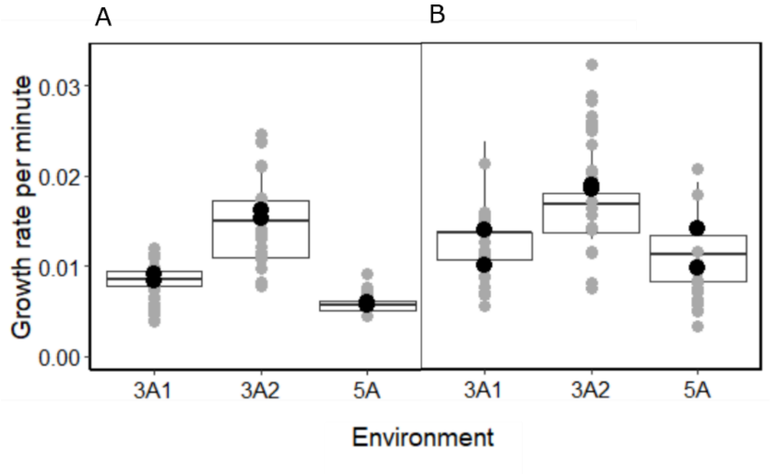

Figure S4: Representative clones for the wild-type and mutator strain evolved in complex evolution environments. Growth rates of eight replicate wild-type (A) and mutator (B) populations (black boxes, vertical axis) in each of the three complex environments (horizontal axis) in which they evolved, at the end of experimental evolution. In each box plot, the thick horizontal line represents the mean growth rate of the eight evolved populations, and the lower and upper boundaries of the box represent the first and the third quartile, respectively. Whiskers show 95% confidence intervals. Circles show the growth rates of 24 clones (three randomly chosen clones from each of the eight populations). Black circles represent the growth rates of the two clones, out of 24, that we selected for novel trait assays and for whole-genome sequencing. We measured all growth rates at the IC_{90} of the respective antibiotic for all populations and clones. Source data are provided as a Source Data file.

Figure S5:

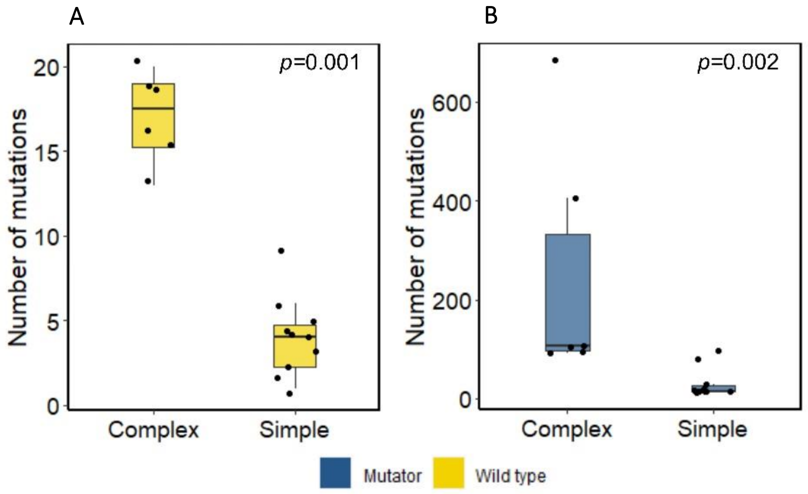

Figure S5: More mutations are retained in complex environments. The number of mutations retained after the experimental evolution is significantly higher for the complex environments compared to the simple environments for both wild-type (panel A, Two-sided Wilcoxon rank sum test, W=0 , n= 6 and 10, *p*=0.001) and mutator (panel B, Two-sided Wilcoxon rank sum test, W=2 , n= 6 and 10, *p*=0.002) strains. The whiskers represent 1.5 times of the interquartile range. The circles located above the top whisker are outliers whose values are higher than 1.5 times the interquartile range (third quartile – first quartile) above the first quartile. Source data are provided as a Source Data file.

Figure S6:

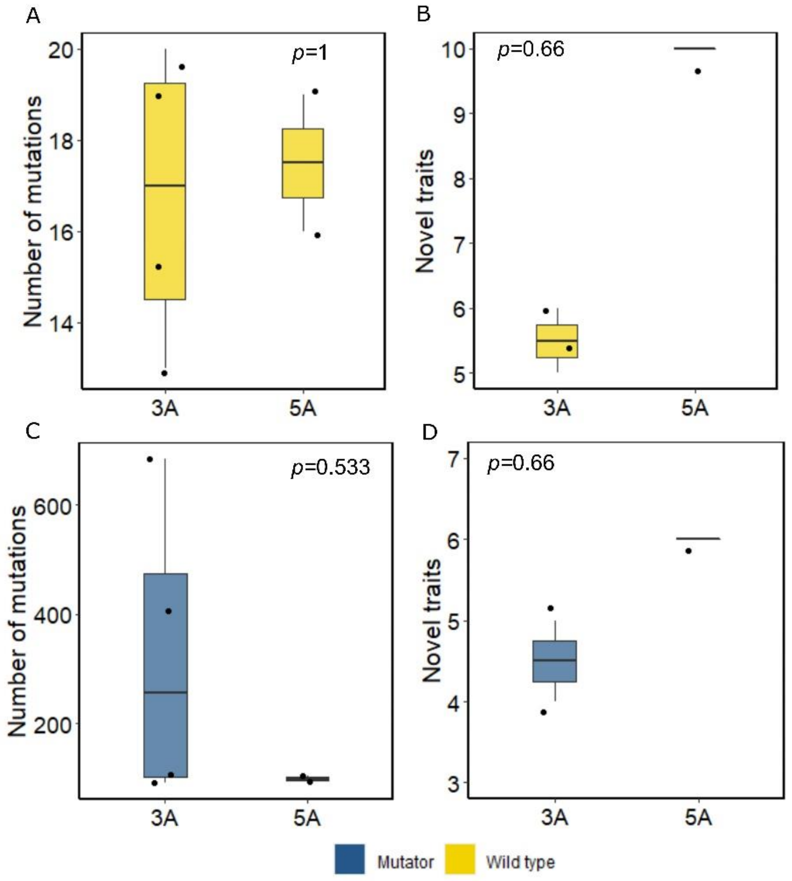

Figure S6: The complexity of the evolution environment, and not the amount of retained genetic variation, affects the extent of novel trait evolution. A. The total number of retained genomic variants was not significantly different between the wild-type clones (yellow) evolved in environments containing three (environments $3A_1$ and $3A_2$) and five (environment 5A) antibiotics (Two-sided Wilcoxon rank sum test, n=4 and 2, W= 3.5, *p*=1). **B.** The number of evolved novel traits was higher but not significantly so for the wild-type clones evolved in the most complex environment (environment 5A) than for clones evolved in environments with intermediate complexity (environments $3A_1$ and $3A_2$) (Two-sided Wilcoxon rank sum test, n=2 and 1, W=0, *p*=0.66). **C.** The total number of retained genomic variants was statistically not distinguishable between the mutator clones (blue) evolved in environments with three (environments $3A_1$ and $3A_2$) and five (environment 5A) antibiotics (Two-sided Wilcoxon rank sum test, n=4 and 2, W=6, *p*=0.533). **D.** The number of evolved novel traits was higher but not significantly so in the mutator clones evolved in the most complex environment (environment 5A) than in clones evolved in environments with intermediate complexity(environments $3A_1$ and $3A_2$) (Two-sided Wilcoxon rank sum test, n=2 and 1, W=0, *p*=0.66,). The midline of the box plots show median while the hinges represent the first and the third quartile and whiskers show the interquartile range. In panel B and D, the box plot on the right represents a single value for novel traits in 5A environments. The circles located above the top whisker are outliers whose values are higher than 1.5 times the interquartile range (third quartile – first quartile) above the first quartile. Source data are provided as a Source Data file.

Supplementary note S3:

We estimated the approximate number of mutations experienced by our mutator populations evolved in the simple environments and our wild-type population evolved in the complex environments. For this purpose, we assumed that every population at the end of 24h growth had grown to $\sim 2\times10^8$ cells, based on pilot experiments that used 2 ml LB broth in 24-well plates. In consequence, 4 µl of serial-transfer inoculum will then contain $\sim 4\times10^5$ cells. The mutator strain has been estimated to experience ~22 mutations per 1000 cell generations [13]. Based on these numbers, $\sim 4\times10^6$ mutational events will occur during 24 hours of growth. We evolved our mutator populations for an average of 17 of such 24 hour rounds in simple environments, which leads to an average of $\sim 7\times10^7$ mutations.

Analogously, wild-type populations evolved in complex environments on average for 145 days to a final daily density of $\sim 2\times10^8$ cells, a serial-transfer inoculum of $\sim 100\times10^5$ cells, and a mutation rate of ~1 mutation per 1000 cell generations, leading to an expected average of $\sim 3\times10^7$ mutations per population.

In sum, the number of mutations experienced by mutator populations evolved in simple environments and wild-type populations evolved in complex environment is of the same order of magnitude.

Table S6: All mutations (second column from left) observed in wild-type clones evolved in multiple antibiotic environments (six clones in total) and mutator clones evolved in single antibiotic environments (ten clones in total). The first column from the left shows the identity of the clone and the environment in which it evolved. Mutations affecting a component of the *AcrAB-TolC* efflux system are marked with ‘ * ‘.

**Strain: Environment (clone)**	**Mutation**	**Gene**	**Mutation target**
Wild-type: $3A_1$ (I)	SNP(I237N)	*dnaK*	Chaperone protein
	SNP(L28R)	*folA*	Dihydrofolate reductase
	SNP(T>C)	*Intergenic region between *acrA* and *acrR*	*acrA* codes for multidrug efflux pump membrane fusion lipoprotein and *acrR* codes for DNA-binding transcriptional repressor
	Large deletion (289 bp)	*valT* and a part of *lysW*	*valT* codes for one of the seven valine t-RNAs and *lysW* codes for one of the six lysine t-RNAs
	SNP(I207N)	*phoQ*	Sensory histidine kinase
	Small insertion (4bp)	Intergenic region between *mdtJ* and *tqsA*	*mdtJ* codes for multidrug/spermidine efflux pump membrane subunit and *tqsA* autoinducer 2 exporter
	SNP(D87N)	*gyrA*	DNA gyrase subunit A
	Small deletion (35bp)	*amiC*	N-acetylmuramoyl-L-alanine amidase C
	Small deletion (1bp)	*yqiH*	Putative fimbrial chaperone
	SNP(A857T)	*malT*	DNA-binding transcriptional activator
	Small insertion (9bp)	*pitA*	Metal phosphate:H(+) symporter
	Small insertion (6bp)	Intergenic region between *fimA* and *fimI*	*fimA* codes for type 1 fimbriae major subunit and *fimI* codes for putative fimbrial protein FimI
	Small deletion (4bp)	*deoC*	Deoxyribose-phosphate aldolase
Wild-type: $3A_1$ (II)	SNP(L28R)	*folA*	Dihydrofolate reductase
	SNP(Y380C)	*ftsI*	Peptidoglycan D,D-transpeptidase
	SNP(T>C)	*Intergenic region between *acrA* and *acrR*	*acrA* codes for multidrug efflux pump membrane fusion lipoprotein and *acrR* codes for DNA-binding transcriptional repressor
	SNP(I207N)	*phoQ*	Sensory histidine kinase
	Small deletion (3bp)	*topA*	DNA topoisomerase 1
	SNP(C111W)	*marR*	DNA-binding transcriptional repressor
	SNP(W85C)	*mlc*	DNA-binding transcriptional repressor
	Small deletion (6bp)	*dcd*	dCTP deaminase
	SNP(D87G)	*gyrA*	DNA gyrase subunit A
	SNP(V44G)	*gyrA*	DNA gyrase subunit A
	Small insertion (9bp)	*pitA*	Metal phosphate:H(+) symporter
	Small insertion (9bp)	*hdfR*	DNA-binding transcriptional dual regulator
	Small deletion (2bp)	*ilvG*	acetolactate synthase II subunit IlvG, N-terminal fragment (pseudogene)
	Small insertion (4bp)	Intergenic region between *cadC* and *pheU*	*cadC* codes for DNA binding transcriptional activator and *pheU* codes for one of the two phenylalanine t-RNA
	SNP(C>G)	Intergenic region between *ampC* and *frdD*	*ampC* codes for beta-lactamase and *frdD* codes for fumarate reductase membrane protein
Wild-type: $3A_2$ (I)	Small insertion (9bp)	*sbmA*	Peptide antibiotic/peptide nucleic acid transporter
	SNP(R717C)	**acrB*	Multidrug efflux pump RND permease
	Small insertion (9bp)	*moaB*	MoaB protein

	Synonymous substitution (A154A)	*cutC*	Protein CutC
	SNP(V243F)	*motA*	Motility protein A
	SNP(A46S)	*fliC*	Flagellar filament structural protein
	SNP(R98C)	*yojI*	ABC transporter family protein/microcin J25 efflux protein
	SNP(D87G)	*gyrA*	DNA gyrase subunit A
	Small deletion (1bp)	*mprA*	DNA-binding transcriptional repressor
	Small insertion (1bp)	*ptsP*	Phosphoenolpyruvate-protein phosphotransferase
	Small insertion (21bp)	*infB*	Translation initiation factor IF-2
	Small insertion (1bp)	*sspA*	Stringent starvation protein A
	Small insertion (1bp)	*rsmG*	16S rRNA m(7)G527 methyltransferase
	Small insertion (1bp)	*rhlB*	ATP-dependent RNA helicase
	Small deletion (2bp)	*cyaA*	Adenylate cyclase
	Small insertion(2bp)	*cyaA*	Adenylate cyclase
	Small deletion (22bp)	Intergenic region between *cpxR* and *cpxP*	*cpxR* codes for DNA-binding transcriptional dual regulator and *cpxP* codes for periplasmic protein
	Small insertion (9bp)	*fimE*	Regulator for fimA
	Small insertion (4bp)	Intergenic region between *fimA* and *fimI*	*fimA* codes for type 1 fimbriae major subunit and *fimI* codes for putative fimbrial protein
Wild-type: 3A_2 (II)	SNP(T>C)	Intergenic region between *ykgM* and *ykgR*	*ykgM* codes for putative ribosomal protein and *ykgR* codes for putative membrane protein
	SNP(G>T)	Intergenic region between *hemB* and *yaiT*	*hemB* codes for porphobilinogen synthase and *yaiT* codes for putative autotransporter
	Small insertion (1bp)	*sbmA*	Peptide antibiotic/peptide nucleic acid transporter
	SNP(L828S)	**acrB*	Multidrug efflux pump RND permease
	Small insertion (9bp)	*moaB*	MoaB protein
	SNP(N502S)	*thrS*	Threonine-tRNA ligase
	Small insertion (6bp) flanked by two small deletions (1bp each) at both ends	*yeaR*	DUF1971 domain-containing protein
	SNP(A>T)	Intergenic region between *fihD* and *insB*	*fihD* codes for DNA-binding transcriptional dual regulator and *insB* codes for IS1 protein
	Small deletion (12bp)	*fimM*	Flagellar motor switch protein
	SNP(*365Y) Synonymous substitution (M1M)	*yejB* *yejE*	*yejB* codes for putative oligopeptide ABC transporter membrane subunit *yejE* codes for putative oligopeptide ABC transporter membrane subunit
	SNP(D87Y)	*gyrA*	DNA gyrase subunit A
	Small deletion (1bp)	*mprA*	DNA-binding transcriptional repressor
	Small insertion (1bp)	*sspA*	Stringent starvation protein A
	SNP(L101F)	*pitA*	Metal phosphate:H(+) symporter
	SNP(P79L)	*rsmG*	16S rRNA m(7)G527 methyltransferase
	Small deletion (2bp)	*cyaA*	Adenylate cyclase
	Small insertion(2bp)	*cyaA*	Adenylate cyclase
	SNP(M158V)	*trkH*	K(+) transporter
	Small deletion (22bp)	Intergenic region between *cpxR* and *cpxP*	*cpxR* codes for DNA-binding transcriptional dual regulator and *cpxP* codes for periplasmic protein CpxP
	Small insertion (1bp)	*proP*	Osmolyte:H(+) symporter
Wild-type: 5A (I)	SNP(F153S)	*folA*	Dihydrofolate reductase
	Small insertion (9bp)	**acrR*	DNA-binding transcriptional repressor
	SNP(E366K)	*ybdR*	Putative Zn(2(+))-dependent alcohol dehydrogenase
	Synonymous substitution (L222L)	*miaB*	Isopentenyl-adenosine A37 tRNA methylthiolase

	SNP(I207N)	*phoQ*	Sensory histidine kinase
	Large deletion (1378bp)	*ynfA*, *ynfB*, part of *speG*	*ynfA* codes for putative transporter, *ynfB* codes for one of two phenylalanine tRNAs and *speG* codes for spermidine N-acetyltransferase
	Synonymous substitution (T70T)	ydiY	Acid-inducible putative outer membrane protein
	SNPs(GG>TC)	Intergenic region between *eco* and *mqo*	*eco* codes for serine protease inhibitor ecotin and *mqo* codes for malate:quinone oxidoreductase
	SNP(F109L)	*gyrA*	DNA gyrase subunit A
	Small deletion (1bp)	*amiC*	N-acetylmuramoyl-L-alanine amidase C
	Large deletion (1752)	*fau, sibC, ibsC*, part of *serA, ssrS*	*fau* codes for putative 5-formyltetrahydrofolate cyclo-ligase*, sibC* codes for small regulatory RNA antitoxin*, ibsC* codes for toxic peptide, *serA* codes for phosphoglycerate dehydrogenase*, ssrS* codes for 6S RNA
	Small insertion (2bp)	*mg*	RNase G
	SNP(R86S)	*rpsL*	30S ribosomal subunit protein S12
	Small deletion (1bp)	*aroK*	Shikimate kinase 1
	SNP(I86S)	*envZ*	Sensory histidine kinase
	Small insertion (9bp)	*pitA*	Metal phosphate:H(+) symporter
	SNP(G>A)	Intergenic region between *mnmG* and *mioC*	*mnmG* codes for 5-carboxymethylaminomethyluridine-tRNA synthase subunit and *mioC* codes for flavoprotein
	Small insertion (9bp)	*ptsA*	Putative PTS multiphosphoryl transfer protein
	Small deletion (11bp)	*pyrB*	Aspartate carbamoyltransferase catalytic subunit
Wild-type: 5A (II)	SNP(W30R)	*folA*	Dihydrofolate reductase
	SNP(E133D)	*rpsB*	30S ribosomal subunit protein S2
	Small deletion (1bp)	*sbmA*	Peptide antibiotic/peptide nucleic acid transporter
	SNP(T5N)	**acrR*	DNA-binding transcriptional repressor
	Large deletion (109bp)	Part of *glnU*	One of the four glutamine tRNAs
	Synonymous substitution (R25R)	*ybjI*	5-amino-6-(5-phospho-D-ribitylamino)uracil phosphatase
	SNP(I207N)	*phoQ*	Sensory histidine kinase
	Small insertion (8bp)	*md*	RNase D
	SNP(D87N)	*gyrA*	DNA gyrase subunit A
	Small deletion (1bp)	Intergenic region between *kgtP* and *rrfG*	*kgtP* codes for alpha-ketoglutarate:H(+) and *rrfG* codes for symporter 5S ribosomal RNA
	SNP(D42E)	*pyrG*	CTP synthetase
	Small deletion (1bp)	*aroK*	Shikimate kinase 1
	SNP(A476D)	*pitA*	Metal phosphate:H(+) symporter
	Small deletion (25bp)	*rsmG*	16S rRNA m(7)G527 methyltransferase
	Small deletion (1bp)	*cyaA*	Adenylate cyclase
	SNP(P1104S)	*rpoB*	RNA polymerase subunit beta
Mutator: amp (I)	SNP(P83S)	*rcsF*	Outer membrane lipoprotein RcsF
	SNP(A210T)	*cnoX*	Chaperedoxin
	SNP(R185H)	*paaY*	2-hydroxycyclohepta-1,4,6-triene-1-carboxyl-CoA thioesterase
	SNP(M1A)	*rsmF*	16S rRNA m(5)C1407 methyltransferase
	SNP(R36H)	*yebZ*	Putative inner membrane protein
	SNP(P12L)	*yfbP*	Uncharacterized protein
	SNP(A397T)	*purF*	Amidophosphoribosyltransferase
	SNP(G259D)	*tsaD*	N(6)-L-threonylcarbamoyladenine synthase subunit
	SNP(D573G)	*fusA*	Elongation factor G

	SNP(A>G)	Intergenic region between *aroK* and *hofQ*	*aroK* codes for shikimate kinase 1 and *hofQ* codes for DNA utilization protein
	Synonymous substitution (G33G)	*xylE*	D-xylose:H(+) symporter
	Small deletion (1 bp)	*frdD*	Fumarate reductase membrane protein
	Small insertion (3bp)	*frdD*	Fumarate reductase membrane protein
	SNP(Y36H)	*fimE*	Regulator for *fimA*
	Synonymous substitution (L192L)	*kefC*	K(+) : H(+) antiporter
	Small insertion (6bp) flanked by two small deletions (1bp each) at both ends	Intergenic region between *ybiX* and *fiu*	*ybiX* codes for PKHD-type hydroxylase and *fiu* codes for putative iron siderophore outer membrane transporter
	SNP(F76L)	*gfcD*	Putative lipoprotein
	Synonymous substitution Q(215Q)	*minD*	Z-ring positioning protein
	Small insertion (1bp)	*trpC*	Fused indole-3-glycerol phosphate synthase/phosphoribosylanthranilate isomerase
	SNP(E112G)	*yneE*	Conserved inner membrane protein
	SNP(A>G)	Intergenic region between *ydgC* and *rstA*	*ydgC* codes for GlpM family protein and *rstA* codes for DNA-binding transcriptional regulator
	SNP(S141P)	*cirA*	Ferric dihyroxybenzoylserine outer membrane transporter
Mutator: amp (II)	Synonymous substitution (L105L)	*yfeH*	Putative solute:Na(+) symporter
	Small insertion (1bp)	*rlmD*	23S rRNA m(5)U1939 methyltransferase
	SNP(P62L)	*bglA*	6-phospho-beta-glucosidase A
	Synonymous substitution (S125S)	*yqiK*	Flotillin family inner membrane protein
	SNP(A695T)	*fusA*	Elongation factor G
	SNP(G164R)	*yhjV*	Putative transporter
	SNP(D393N)	*cadC*	DNA-binding transcriptional activator
	SNP(T118I)	*frdD*	Fumarate reductase membrane protein
	SNP(S34N)	*cpdB*	2'3' cyclic nucleotide phosphodiesterase/3' nucleotidase
	SNP(G161E)	*fimE*	Regulator for fimA
	SNP(L55P)	*yadN*	Putative fimbrial protein
	SNP(D263N)	*yegS*	Lipid kinase
	SNP(E138G)	*menH*	2-succinyl-6-hydroxy-2, 4-cyclohexadiene-1-carboxylate synthase
	SNP(A>G)	Intergenic region between *yfdI* and *yfdK*	*yfdI* codes for a part of CPS-53 (KpLE1) prophage and homologous with serotype specific glucosyl transferase and *yfdK* codes for a part of CPS-53 (KpLE1) prophage and homologous with putative tail fiber assembly protein
	Synonymous substitution (I371I)	*yphH*	Putative DNA-binding transcriptional regulator, NAGC-like
	SNP(W13R)	*ygjG*	Glutathionyl-hydroquinone reductase
Mutator: azi (I)	SNP(G66S)	*rplD*	50S ribosomal subunit protein L4
	SNP(A667V)	*fusA*	Elongation factor G
	SNP(Q122R)	*fusA*	Elongation factor G
	Synonymous substitution (A555A)	*yhfK*	Putative transporter
	SNP(C182R)	*bcsB*	Cellulose synthase periplasmic subunit
	Small insertion (9bp)	*sgbU*	Putative L-xylulose 5-phosphate 3-epimerase
	Small insertion (1bp)	*rpmH*	50S ribosomal subunit protein L34
	Synonymous substitution (S291S)	*dusA*	tRNA-dihydrouridine synthase A
Mutator: azi (II)	SNP(A325P)	*surA*	Peptidyl-prolyl cis-trans isomerase
	Synonymous substitution (E44E)	*mhpB*	3-carboxyethylcatechol 2,3-dioxygenase

	SNP(W68*)	*dgcC*	Diguanylate cyclase
	SNP(C>T)	pseudogene *efeU*	Inactive ferrous iron permease
	Small deletion (1bp)	Intergenic region between *iraM* and *ymgK*	*iraM* codes for anti-adaptor protein and *ymgK* codes for inhibitor of sigma(S) proteolysis
	SNP (R94C)	*marR*	DNA-binding transcriptional repressor
	SNP(A>G)	Intergenic region between *chbF* and *chbR*	*chbF* codes for monoacetylchitobiose-6-phosphate hydrolase and *chbR* codes for DNA-binding transcriptional dual regulator
	SNP(D462G)	*recC*	exodeoxyribonuclease V subunit
	Small insertion (1bp)	*garK*	glycerate 2-kinase 1
	SNP(Q250*)	*agaC*	Galactosamine-specific PTS enzyme IIC component
	SNP(F605S)	*fusA*	Elongation factor G
	Synonymous substitution (S352S)	*cysG*	Siroheme synthase
	SNP(D427G)	*bcsA*	Cellulose synthase catalytic subunit
	SNP(H163Y)	*yieP*	Putative transcriptional regulator
Mutator: nal (I)	SNP(A210T)	*cnoX*	Chaperedoxin
	SNP(T370A)	*gfcE*	Putative exopolysaccharide export lipoprotein
	SNP(H115R)	*insB-4*	IS1 protein
	SNP(R185H)	*paaY*	2-hydroxycyclohepta-1,4,6-triene-1-carboxyl-CoA thioesterase
	SNP(D87G)	*gyrA*	DNA gyrase subunit A
	SNP(A397T)	*purF*	Amidophosphoribosyltransferase
	Small insertion (1bp)	Intergenic region between *fadL* and *yfdF*	*fadL* codes for long-chain fatty acid outer membrane channel *yfdF* codes for bacteriophage T2 receptor
	SNP(T>C)	Intergenic region between *ypjC* and *ileY*	*ypjC* codes for DUF5507 domain-containing protein and *ileY* codes for one of the five isoleucine tRNAs
	SNP(G259D)	*tsaD*	N(6)-L-threonylcarbamoyladenine synthase
	Small insertion (1bp)	*frlD*	Fructoselysine 6-kinase
	Synonymous substitution (D193D)	*sgbU*	Putative L-xylulose 5-phosphate 3-epimerase
	Small insertion (1bp)	Intergenic region between *ilvL* and *ilvX*	*ilvL* codes for operon leader peptide and *ilvX* codes for uncharacterized protein
	SNP(Y36H)	*fimE*	Regulator of *fimA*
Mutator: nal (II)	SNP(F116L)	*rapA*	RNA polymerase-binding ATPase and RNAP recycling factor
	SNP(T126A)	*yafP*	Putative acyltransferase with acyl-CoA N-acyltransferase domain
	Synonymous substitution (A272A)	*yahE*	DUF2877 domain-containing protein
	SNP(V32A)	*yccU*	Putative CoA-binding protein with NAD(P)-binding Rossmann-fold domain
	SNP(V67A)	*tusE*	Sulfur transfer protein
	SNP(T>C)	Intergenic region between *serT* and *hyaA*	*serT* codes for one of the five serine tRNAs and *hyaA* codes for hydrogenase 1 small subunit
	SNP(V103A)	*ydcI*	Putative DNA-binding transcriptional repressor
	Synonymous substitution (L110L)	*nhoA*	Arylamine N-acetyltransferase
	SNP(N9I)	*yddK*	Leucine-rich repeat domain-containing protein
	SNP(C>A)	*valW*	One of the seven valine t-RNAs
	SNP(D87G)	*gyrA*	DNA gyrase subunit A
	SNP(H9L)	*yfbR*	dCMP phosphohydrolase
	SNP(R612*)	*fadJ*	3-hydroxyacyl-CoA dehydrogenase FadJ
	SNP(V186A)	*eutR*	Putative AraC-type transcriptional regulator
	SNP(M267V)	*eutG*	Putative alcohol dehydrogenase in ethanolamine utilization
	SNP(D97G)	*lgt*	Phosphatidylglycerol--prolipoprotein diacylglyceryl transferase
	SNP(T69A)	*yghD*	Putative type II secretion system M-type protein

	Small insertion (1bp)	*yqiA*	Esterase
	SNP(A455T)	*garD*	Galactarate dehydratase
	SNP(T630A)	*ftsH*	ATP-dependent zinc metalloprotease
	SNP(G612S)	*yheS*	ABC transporter ATP-binding protein
	SNP(S574G)	*bcsA*	Cellulose synthase catalytic subunit
	SNP(T456A)	*ilvA*	Threonine deaminase
	Synonymous substitution (K17K)	*argH*	Argininosuccinate lyase
	SNP(W160*)	*qorA*	Putative quinone oxidoreductase 1
	SNP(A8T)	*mdtN*	Putative multidrug efflux pump membrane fusion protein
	Synonymous substitution (249L)	*yjhI*	KpLE2 phage-like element, putative DNA-binding transcriptional regulator
	SNP(T8A)	*fimE*	Regulator of *fimA*
	Small insertion (1bp)	*leuV*	One of the eight leucine tRNAs
Mutator: strep (I)	Synonymous substitution (V51V)	*ivy*	Periplasmic chaperone, inhibitor of vertebrate C-type lysozyme
	Small deletion (1bp)	Intergenic region between *frmR* and *yaiO*	*frmR* codes for DNA-binding transcriptional repressor and *yaiO* codes for outer membrane protein
	SNP(F298L)	*mdlA*	ABC transporter family protein
	SNP(A210T)	*cnoX*	Chaperedoxin
	SNP(R185H)	*paaY*	2-hydroxycyclohepta-1,4,6-triene-1-carboxyl-CoA thioesterase
	SNP(A>G)	Intergenic region between *ydfJ* and *ynfT*	*ydfJ* codes for putative transporter and *ynfT* codes for qin prophage protein
	SNP(Q2E)	*araG*	Arabinose ABC transporter ATP binding subunit
	SNP(L28P)	*udk*	Uridine/cytidine kinase
	SNP(P12L)	*yfbP*	Uncharacterized protein
	SNP(A397T)	*purF*	Amidophosphoribosyltransferase
	SNP(A731T)	*gcvP*	Glycine decarboxylase
	SNP(G259D)	*tsaD*	N(6)-L-threonylcarbamoyladenine synthase
	Synonymous substitution (G5G)	*zntR*	DNA-binding transcriptional activator
	SNP(K88R)	*rpsL*	30S ribosomal subunit protein S12
	SNP(Y36H)	*fimE*	Regulator of *fimA*
	SNP(E38G)	*fimA*	Type 1 fimbriae major subunit
Mutator: strep (II)	SNP(C>T)	Intergenic region between *pepD* and *gpt*	*pepD* codes for peptidase D and *gpt* codes for xanthine-guanine phsophoribosyltransferase
	SNP(Q554*)	*ycaO*	Ribosomal protein S12 methylthiotransferase accessory factor
	SNP(P242L)	*lpxK*	Tetraacyldisaccharide 4'-kinase
	SNP(D161N)	*yccE*	Uncharacterized protein
	SNP(T>C)	Intergenic region between *pgrR* and *mppA*	*pgrR* codes for DNA-binding transcriptional repressor and *mppA* codes for murein tripeptide ABC transporter periplasmic binding protein
	SNP(T>C)	Intergenic region between *araF* and *ftnB*	*araf* codes for arabinose ABC transporter periplasmic binding protein and *ftnB* codes for putative ferritin-like protein
	Small deletion (1bp)	Intergenic region between *yqcW* and yqcE	*yqcW* codes for putative deoxygluconate dehydrogenase and *yqcE* codes for putative transport protein
	SNP(N26S)	*ygcE*	Putative sugar kinase
	SNP(K88R)	*rpsL*	30S ribosomal subunit protein S12
	SNP(A>G)	Intergenic region between *glvC* and *yidP*	*glvC* codes for putative PTS enzyme II component and *yidP* codes for putative DNA-binding transcriptional regulator
	SNP(N175D)	*fimE*	Regulator of *fimA*
Mutator: tri (I)	Small deletion (1bp)	Intergenic region between *thrL* and *thrA*	*thrL* codes for thr operon leader peptide and *thrA* codes for fused aspartate kinase/homoserine dehydrogenase 1
	SNP(G>A)	Intergenic region between *kefC* and *folA*	*kefC* codes for K(+) : H(+) antiporter and *folA* codes for dihydrofolate reductase

	SNP(P21L)	*folA*	Dihydrofolate reductase
	Synonymous substitution (G213G)	*pdxA*	4-hydroxythreonine-4-phosphate dehydrogenase
	SNP(I342F)	*polB*	DNA polymerase II
	Small deletion (1bp)	*fhuB*	Iron(III) hydroxamate ABC transporter membrane subunit
	Small deletion (1bp)	*rrsH*	16S ribosomal RNA
	SNP(E49G)	*rayT*	REP-associated tyrosine transposase
	Small deletion (1bp)	*decR*	DNA-binding transcriptional activator
	SNP(Q75*)	*mdlB*	ABC transporter family protein
	SNP(E203G)	*gsk*	Inosine/guanosine kinase
	Small deletion (2bp)	*rna*	RNase I
	Synonymous substitution (S14S)	*insH-3*	IS5 transposase and trans-activator
	SNP(C264R)	*nagC*	DNA-binding transcriptional dual regulator
	SNP(T>A)	pseudogene *ybfL*	Putative transposase
	SNP(V519A)	*mngB*	Alpha-mannosidase
	SNP(T39A)	*tolQ*	Tol-Pal system protein
	SNP(D164E)	*nadA*	Quinolinate synthase
	SNP(I350V)	*modC*	Molybdate ABC transporter ATP binding subunit
	Synonymous substitution(G315G)	*dacC*	D-alanyl-D-alanine carboxypeptidase
	Synonymous substitution (G60G)	*ycaO*	Ribosomal protein S12 methylthiotransferase accessory factor
	SNP(F23L)	*msbA*	ATP-binding lipopolysaccharide transport protein
	SNP(I91N)	*etk*	Protein-tyrosine kinase
	Synonymous substitution (G429G)	*torA*	Trimethylamine N-oxide reductase 1
	SNP(K122E)	*dhaL*	Dihydroxyacetone kinase subunit L
	Synonymous substitution (G228G)	*dauA*	Aerobic C4-dicarboxylate transporter
	SNP(S119L)	*tonB*	Ton complex subunit
	SNP(I33N)	*ycjC*	Putative inner membrane protein
	SNP(H228Q)	*ycjW*	Putative LacI-type DNA-binding transcriptional regulator
	SNP(T14P)	*tyrR*	DNA-binding transcriptional dual regulator
	SNP(Y374C)	*ydcS*	Putative ABC transporter periplasmic binding protein /polyhydroxybutyrate synthase
	Synonymous substitution (A65A)	*ydcY*	DUF2526 domain-containing protein
	Small deletion (1bp)	pseudogene *yneO*	AIDA-I family autotransporter
	SNP(G>A)	pseudogene *yneO*	AIDA-I family autotransporter
	SNP(G>A)	Intergenic region *ynfC* and *ynfD*	*ynfC* codes for UPF0257 family lipoprotein and *ynfD* codes for DUF1161 domain-containing protein
	SNP(T47A)	*ynfK*	Putative dethiobiotin synthetase
	Small insertion (1bp)	*ydhJ*	Putative membrane fusion protein
	Small deletion (1bp)	*ydhV*	Putative oxidoreductase
	SNP(A120G)	*dmlA*	D-malate/3-isopropylmalate dehydrogenase (decarboxylating)
	SNP(E28G)	*alkA*	DNA-3-methyladenine glycosylase 2
	SNP(T198A)	*folE*	GTP cyclohydrolase 1
	Synonymous substitution (R630R)	*cirA*	Ferric dihyroxybenzoylserine outer membrane transporter
	SNP(S120P)	*folC*	Bifunctional folylpolyglutamate synthetase/dihydrofolate synthetase
	SNP(T18A)	*mnmC*	Fused 5-methylaminomethyl-2-thiouridine-forming methyltransferase and FAD-dependent demodification enzyme

	Small deletion (1bp)	Intergenic region between *fadL* and *yfdF*	*fadL* codes for long-chain fatty acid outer membrane channel and *yfdF* codes for bacteriophage T2 receptor/protein YfdF
	Small deletion (1bp)	*yfdE*	*yfdE* codes for acetyl-CoA:oxalate CoA-transferase
	SNP(F324L)	*ppk*	Polyphosphate kinase
	SNP(F26S)	*sseB*	Protein SseB
	SNP(A>T)	*ryfD*	Small regulatory RNA
	SNP(T60A)	*argP*	DNA-binding transcriptional dual regulator
	Small deletion (1bp)	Intergenic region between *glcA* and *glcB*	*glcA* codes for glycolate/lactate:H(+) symporter and *glcB* codes for malate synthase G
	Synonymous substitution (L372L)	*hybB*	Hydrogenase 2 membrane subunit
	SNP(P19S)	*mqsA*	Antitoxin of the MqsRA toxin-antitoxin system/DNA-binding transcriptional repressor
	Synonymous substitution (D812D)	*yraJ*	Putative fimbrial usher protein
	SNP(D66E)	*rimP*	Ribosome maturation factor
	SNP(D124E)	*zapE*	Cell division factor ZapE with ATPase activity
	Small deletion (1bp)	*mg*	RNase G
	SNP(T>C)	Intergenic region between *rpsJ* and *gspB*	*rpsJ* codes for 30S ribosomal subunit protein S10 and *gspB* codes for putative general secretion pathway protein B
	SNP(D254N)	*yheT*	Putative hydrolase
	SNP(I159F)	*yhfK*	Putative transporter
	Small deletion (1bp)	*dam*	DNA adenine methyltransferase
	SNP(S11P)	*ftsY*	Signal recognition particle receptor
	Small deletion (1bp)	*yhhH*	PF15631 family protein
	Small deletion (1bp)	*yhhJ*	ABC transporter family protein
	SNP(D566E)	*mdtF*	Multidrug efflux pump RND permease
	Small insertion (1bp)	Intergenic region between *bcsG* and *ldrD*	*bcsG* codes for cellulose phosphoethanolamine transferase and *ldrD* codes for small toxic polypeptide
	Small deletion (1bp)	*wecH*	O-acetyltransferase
	SNP(S288P)	*yiaN*	2,3-diketo-L-gulonate:Na(+) symporter - membrane subunit
	SNP(D422E)	*gpmM*	2,3-bisphosphoglycerate-independent phosphoglycerate mutase
	SNP(K273E)	*dnaA*	Chromosomal replication initiator protein
	SNP(T135A)	*pstB*	Phosphate ABC transporter ATP binding subunit
	SNP(I318T)	*glnL*	Sensory histidine kinase NtrB
	Synonymous substitution (L579L)	*frvR*	Putative transcriptional regulator
	SNP(N290D)	*metA*	Homoserine O-succinyltransferase
	SNP(A>G)	Intergenic region between *rluF* and *yjbD*	*rluF* codes for 23S rRNA pseudouridine(2604) and tRNA(Tyr) pseudouridine(35) synthase and *yjbD* is a conserved protein
	SNP(N128S)	*phnF*	Putative transcriptional regulator
	SNP(A46T)	*cpdB*	2'3' cyclic nucleotide phosphodiesterase/3' nucleotidase
	SNP(I193N)	*mgtA*	Mg(2(+)) importing P-type ATPase
	SNP(A>G)	Intergenic region between *nanC* and *fimB*	*nanC* codes for N-acetylneuraminic acid outer membrane channel and *fimB* codes for regulator for fimA
	Synonymous substitution (L100L)	*yjiQ*	DNA-binding transcriptional repressor
	SNP(I343N)	*deoB*	Phosphopentomutase
Mutator: tri (II)	SNP(I5F)	*folA*	Dihydrofolate reductase
	SNP(F153S)	*folA*	Dihydrofolate reductase
	Synonymous substitution (G213G)	*pdxA*	4-hydroxythreonine-4-phosphate dehydrogenase
	SNP(I342F)	*polB*	DNA polymerase II
	SNP(I103V)	*fhuB*	Iron(III) hydroxamate ABC transporter membrane subunit

	SNP(I97V)	*bamA*	Outer membrane protein assembly factor
	SNP(K247T)	*yaeF*	Peptidase C92 family protein
	SNP(I326F)	*ecpD*	Fimbrial adhesin
	SNP(N3T)	*mhpR*	DNA-binding transcriptional activator
	Synonymous substitution (R497R)	*sbcC*	ATP dependent, structure specific DNA nuclease
	SNP(D278G)	*queA*	tRNA preQ1(34) S-adenosylmethionine ribosyltransferase-isomerase
	Small insertion (1bp)	Intergenic region between *queA* and *tgt*	tRNA preQ1(34) S-adenosylmethionine ribosyltransferase-isomerase/tRNA-guanine transglycosylase
	Synonymous substitution (G41G)	*yajL*	Protein/nucleic acid deglycase 3
	Small insertion (1bp)	*yajQ*	Nucleotide binding protein
	Synonymous substitution (S484S)	*cyoB*	Cytochrome bo3 ubiquinol oxidase subunit 1
	SNP(Q75*)	*mdlB*	ABC transporter family protein
	Synonymous substitution (S298S)	*mscK*	Potassium dependent, small conductance mechanosensitive channel
	SNP(D19G)	*ybcK*	DLP12 prophage, putative recombinase
	SNP(C>T)	pseudogene *ybcY*	DLP12 prophage, putative SAM-dependent methyltransferase
	Synonymous substitution (D59D)	*ybfF*	Esterase
	SNP(N245D)	*cydB*	Cytochrome bd-I ubiquinol oxidase subunit II
	SNP(S76P)	*sxy*	Transcriptional coactivator for CRP
	SNP(A431T)	*rne*	Ribonuclease E
	Synonymous substitution (L130L)	*yceF*	m(7)GTP pyrophosphatase
	Synonymous substitution (G76G)	*ldtC*	L,D-transpeptidase YcfS
	SNP(D158G)	*roxA*	Ribosomal protein-arginine oxygenase
	SNP(I109T)	*phoQ*	Sensory histidine kinase
	SNP(K166E)	*hflD*	Lysogenization regulator
	Small deletion (1bp)	*dhaM*	Dihydroxyacetone kinase subunit M
	SNP(S119L)	*tonB*	Ton complex subunit
	Synonymous substitution (V56V)	*yciB*	Inner membrane protein
	Small insertion (1bp)	*topA*	DNA topoisomerase 1
	SNP(V135A)	*ycjR*	Putative ketohexose-3-epimerase
	SNP(D51G)	*curA*	NADPH-dependent curcumin/dihydrocurcumin reductase
	SNP(V263A)	*ydeQ*	Putative fimbrial adhesin protein
	Small insertion (1bp)	pseudogene *yneQ*	AIDA-I family autotransporter
	SNP(S11P)	*pinQ*	Qin prophage, putative site-specific recombinase
	SNP(T47A)	*ynfK*	Putative dethiobiotin synthetase
	Synonymous substitution (E11E)	*nemR*	DNA-binding transcriptional repressor
	SNP(D186V)	*ydjB*	Shikimate dehydrogenase/quinate dehydrogenase
	SNP(D558G)	*fadD*	Fatty acyl-CoA synthetase
	SNP(T47A)	*eda*	KHG/KDPG aldolase
	Synonymous substitution (D288D)	*yebK*	DNA-binding transcriptional repressor
	SNP(F316S)	*wbbI*	Beta-1,6-galactofuranosyltransferase
	SNP(K349E)	*wcaD*	Putative colanic acid polymerase
	SNP(G>A)	Intergenic region between *baeR* and *yegP*	*baeR* codes for DNA-binding transcriptional activator and *yegP* codes for DUF1508 domain-containing protein YegP
	SNP(T198A)	*folE*	GTP cyclohydrolase 1

	Small deletion (1bp)	*yejB*	Putative oligopeptide ABC transporter membrane subunit
	Small insertion (1bp)	*elaD*	Protease
	SNP(K34E)	*intS*	CPS-53 (KpLE1) prophage, prophage CPS-53 integrase
	SNP(I84V)	*cysU*	Sulfate/thiosulfate ABC transporter inner membrane subunit
	SNP(A24T)	*murP*	N-acetylmuramic acid-specific PTS enzyme IICB component/anhydro-N-acetylmuramic acid transporter
	Synonymous substitution (C60C)	*tmcA*	tRNA(Met) cytidine acetyltransferase
	SNP(D188G)	*hda*	Inibitor of reinitiation of DNA replication
	SNP(A97V)	*glnB*	Nitrogen regulatory protein PII-1
	SNP(A>G)	pseudogene *yfjV*	CP4-57 prophage, putative arsenite transporter
	Small deletion (1bp)	*ypjB*	DUF5508 domain-containing protein
	SNP(I108F)	*nrdF*	Ribonucleoside-diphosphate reductase 2 subunit beta
	Small insertion (1bp)	*xdhA*	Putative xanthine dehydrogenase molybdenum-binding subunit
	Small deletion (1bp)	pseudogene *yghE*	Putative type II secretion system L-type protein
	Small insertion (1bp)	*ygjQ*	Radical SAM superfamily protein
	SNP(P19S)	*mqsA*	Antitoxin of the MqsRA toxin-antitoxin system/DNA-binding transcriptional repressor
	SNP(F145S)	*pnp*	Polynucleotide phosphorylase
	SNP(V368A)	*nanT*	N-acetylneuraminate:H(+) symporter
	SNP(E65G)	*rpsE*	30S ribosomal subunit protein S5
	SNP(E161G)	*rpsC*	30S ribosomal subunit protein S3
	SNP(E699G)	*nirB*	Nitrite reductase catalytic subunit
	SNP(I627V)	*igaA*	Inner membrane protein - inhibits the Rcs signaling pathway
	Synonymous substitution (S254S)	*glgA*	Glycogen synthase
	SNP(A>G)	Intergenic region between *yhhY* and *yhhZ*	*yhhY* codes for N-acetyltransferase and *yhhZ* codes for putative endonuclease
	SNP(I69N)	*nikA*	Ni(2(+)) ABC transporter periplasmic binding protein
	SNP(T>C)	Intergenic region between *nikR* and *rhsB*	*nikR* codes for DNA-binding transcriptional repressor and *rhsB* codes for rhs element protein
	SNP(C469C)	*pdeK*	Putative c-di-GMP phosphodiesterase
	SNP(H231R)	*yiaQ*	2,3-diketo-L-gulonate:Na(+) symporter - periplasmic binding protein
	SNP(T>A)	Intergenic region between *waaQ* and *waaA*	*waaQ* codes for lipopolysaccharide core heptosyltransferase 3 and *waaA* codes for KDO transferase
	SNP(V301A)	*ligB*	DNA ligase B
	Synonymous substitution (D122D)	*gltS*	Glutamate:sodium symporter
	Small deletion (1bp)	*yicL*	Putative inner membrane protein
	SNP(T>A)	*rrlC*	23S ribosomal RNA
	Small deletion (1bp)	*hdfR*	DNA-binding transcriptional dual regulator
	SNP(I205V)	*rffG*	dTDP-glucose 4,6-dehydratase 2
	SNP(S172P)	*pldA*	Outer membrane phospholipase A
	Small deletion (1bp)	Intergenic region between *fre* and *fadA*	*fre* codes for flavin reductase and *fadA codes for* 3-ketoacyl-CoA thiolase
	SNP(Y112S)	*sbp*	Sulfate/thiosulfate ABC transporter periplasmic binding protein
	SNP(A>G)	Intergenic region between *thrT* and *tufB*	*thrT* codes for one of the four threonine tRNAs and *tufB* codes for translation elongation factor Tu 2
	SNP(S386P)	*aceK*	Isocitrate dehydrogenase kinase/phosphatase
	Synonymous substitution (P902P)	*metH*	Cobalamin-dependent methionine synthase
	Synonymous substitution (I239I)	*ycjE*	Putative transporter

	Small deletion (1bp)	*acs*	Acetyl-CoA synthetase (AMP-forming)
	SNP(Y368C)	*mdtO*	Putative multidrug efflux pump subunit
	SNP(S182P)	*alsR*	DNA-binding transcriptional repressor
	SNP(F870S)	*mgtA*	Mg(2(+)) importing P-type ATPase
	SNP(D14E)	*yihU*	KpLE2 phage-like element, putative DNA-binding transcriptional regulator
	Small deletion (1bp)	*hsdR*	Type I restriction enzyme EcoKI endonuclease component
	SNP(T>A)	Intergenic region between *opqB* and *yjjA*	*opqB* codes for phosphoglycerol transferase I and *yjjA* codes for DUF2501 domain-containing protein
	Synonymous substitution (L100L)	*yjjQ*	DNA-binding transcriptional repressor
	Small insertion (1bp)	*leuV*	One of the eight leucine t-RNAs
	Small insertion (1bp)	*leuP*	One of the eight leucine t-RNAs

Table S7:

The number of evolved novel traits (third column from the left) and number of mutations retained by the two clones (fourth column from the left) from the wild-type and mutator strains (first column from the left) evolved in simple and complex environments (second column from the left).

Strain	Environment	Number of evolved novel traits	Number of mutations
Wild-type	Ampicillin	4	1, 2
Wild-type	Azithromycin	2	2, 4
Wild-type	Nalidixic acid	2	6, 3
Wild-type	Streptomycin	3	4, 4
Wild-type	Trimethoprim	1	9, 5
Wild-type	$3A_1$	6	13, 15
Wild-type	$3A_2$	5	19, 20
Wild-type	5A	10	19, 16
Mutator	Ampicillin	3	14, 19
Mutator	Azithromycin	2	14, 14
Mutator	Nalidixic acid	1	13, 29
Mutator	Streptomycin	4	16, 11
Mutator	Trimethoprim	0	81, 98
Mutator	$3A_1$	5	684, 406
Mutator	$3A_2$	4	107, 91
Mutator	5A	6	104, 94

Table S8: Mutations in *AcrAB-TolC* genes for mutator clones from the $3A_1$, $3A_2$ and 5A environments. Identifying other candidate mutations for cellular targets of antibiotics and for multi-drug resistance reliably was infeasible in these mutator clones, because they accumulated hundreds of mutations (Table S7).

**Strain: Environment (clone)**	**Mutation**	**Gene**	**Mutation target**
Mutator: $3A_1$ (I)	Synonymous substitution(K354K)	*acrA*	Multidrug efflux pump membrane fusion lipoprotein
	Synonymous substitution(F280F)	*acrA*	Multidrug efflux pump membrane fusion lipoprotein
	SNP(T>C)	Intergenic region between *acrA* and *acrR*	*acrA* codes for multidrug efflux pump membrane fusion lipoprotein and *acrR* codes for DNA-binding transcriptional repressor
	SNP(A54V)	*acrD*	Multidrug efflux pump RND permease
Mutator: $3A_1$ (II)	SNP(T>C)	Intergenic region between *acrA* and *acrR*	*acrA* codes for multidrug efflux pump membrane fusion lipoprotein and *acrR* codes for DNA-binding transcriptional repressor
	Small insertion (1bp)	*acrF*	Multidrug efflux pump RND permease
Mutator: $3A_2$ (I)	SNP(R717H)	*acrB*	Multidrug efflux pump RND permease
	SNP(A>G)	Intergenic region between *acrA* and *acrR*	*acrA* codes for multidrug efflux pump membrane fusion lipoprotein and *acrR* codes for DNA-binding transcriptional repressor
Mutator: $3A_2$ (I)	SNP(R717C)	*acrB*	Multidrug efflux pump RND permease
	Small deletion (1bp)	Intergenic region between *acrA* and *acrR*	*acrA* codes for multidrug efflux pump membrane fusion lipoprotein and *acrR* codes for DNA-binding transcriptional repressor
Mutator: 5A (I)	SNP(A>G)	Intergenic region between *acrA* and *acrR*	*acrA* codes for multidrug efflux pump membrane fusion lipoprotein and *acrR* codes for DNA-binding transcriptional repressor
	SNP(Q125*)	*acrF*	Multidrug efflux pump RND permease
Mutator: 5A (I)	SNP(L828S)	*acrB*	Multidrug efflux pump RND permease
	SNP(L377P)	*acrB*	Multidrug efflux pump RND permease
	SNP(A39T)	*acrB*	Multidrug efflux pump RND permease
	SNP(T5A)	*acrR*	DNA-binding transcriptional repressor

Table S9: Stock solutions of antibiotics used for experimental evolution

Antibiotic	Solvent	Stock concentration
Trimethoprim	99% water + 1% glacial acetic acid	10 mg/ml
Azithromycin	Absolute ethanol	50 mg/ml
Streptomycin	Water	50 mg/ml
Ampicillin	99.5% water + 0.05% HCl	10 mg/ml
Nalidixic acid	99% water + 1% 10M NaOH	10 mg/ml

Figure S7:

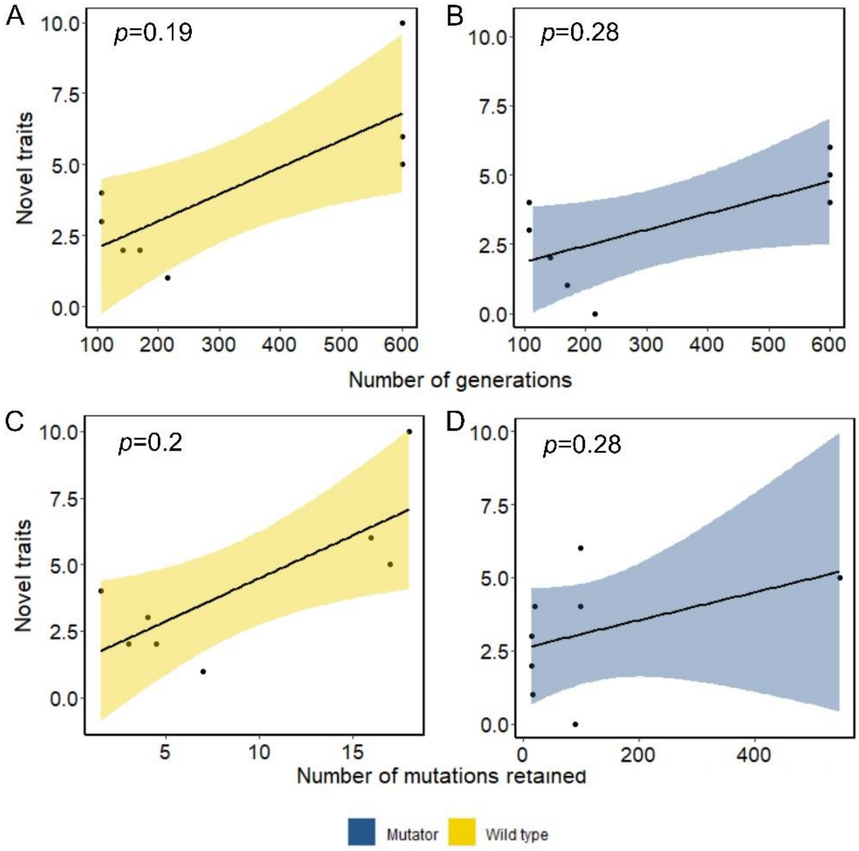

Figure S7: Mutation supply does not affect the evolution of novel traits without immediate benefits. A and B. The number of novel traits (y-axis) is not correlated with the number of generations a population spent in the evolution environment (a proxy of mutation supply) for the evolved wild-type (panel A, Spearman's correlation, n=8, R=0.52, *p*=0.19) and the mutator (panel B, Spearman's correlation, n=8, R=0.44, *p*=0.28) clones. Each black circle represents results from one antibiotic environment. Each number of generations corresponding to a circle is an average over the eight replicate populations evolved in the respective environment. The number of novel traits is based on the two clones analyzed from each environment. **C and D.** The number of novel traits (y-axis) is not correlated with the number of mutations retained after evolution for the evolved wild-type (panel A, Spearman's correlation, n=8, R=0.5, *p*=0.2) and for the mutator (panel B, Spearman's correlation, n=8, R=0.44, *p*=0.28) clones. Each value of a number of retained mutations is an average of the two sequenced clones that evolved in a particular environment. The shaded region represents the 95% confidence intervals. Source data are provided as a Source Data file.

Table S10: Antimicrobials on which viability evolved belonged to a broad diversity of drug-classes whose mechanism of action generally differs from that of the antibiotic in the evolution environment. Five of the seven drug-classes used in the classification of the mechanism of action share this mechanism with the five antibiotics used during our evolution experiment. Antimicrobials with a mechanism of action different from these five antibiotics are in the class ‘other’, while antimicrobials with an unknown mechanism of action are grouped in the class ‘unknown’. Antimicrobials that belong to a drug-class different than the antimicrobial in the evolution environment are marked with an ‘*’.

Evolution environment	Strain	Antimicrobial on which viability evolved	Drug-class of the antimicrobial on which viability evolved
Ampicillin	Wild-type	Chlorpromazine	Ampicillin-like
		Cinnamic acid	Ampicillin-like
		Gallic acid	Ampicillin-like
		Thiamphenicol*	Azithromycin-like
Azithromycin	Wild-type	Josamycin	Azithromycin-like
		Minocycline*	Streptomycin-like
Nalidixic acid	Wild-type	Gallic acid*	Ampicillin-like
		Minocycline*	Streptomycin-like
Streptomycin	Wild-type	4-aminopyridine*	Other
		Phenylarsine oxide*	Other
		Thiamphenicol*	Azithromycin-like
Trimethoprim	Wild-type	Thiamphenicol*	Azithromycin-like
$3A_1$	Wild-type	4-aminopyridine*	Other
		D,L-Thioctic acid	Ampicillin-like
		Josamycin*	Azithromycin-like
		Minocycline*	Streptomycin-like
		Phenylarsine oxide*	Other
		Thiamphenicol*	Azithromycin-like
$3A_2$	Wild-type	Josamycin	Azithromycin-like
		Minocycline	Streptomycin-like
		Puromycin	Streptomycin-like
		Spectinomycin	Streptomycin-like
		Thiamphenicol	Azithromycin-like
5A	Wild-type	Amitriptyline*	Unknown
		Chlorpromazine	Ampicillin-like
		Cobalt chloride*	Unknown
		Josamycin	Azithromycin-like
		Minocycline	Streptomycin-like
		Phenethicillin	Ampicillin-like
		Pridinol*	Unknown
		Promethazine	Ampicillin-like
		Puromycin	Streptomycin-like
		Thiamphenicol	Azithromycin-like
Ampicillin	Mutator	D,L-Thioctic acid	Ampicillin-like
		Puromycin*	Streptomycin-like
		Spectinomycin*	Streptomycin-like
Azithromycin	Mutator	Josamycin	Azithromycin-like
		Thiamphenicol	Azithromycin-like
Nalidixic acid	Mutator	Thiamphenicol*	Azithromycin-like
Streptomycin	Mutator	4-aminopyridine*	Other
		Josamycin*	Azithromycin-like
		Phenylarsine oxide*	Other
		Thiamphenicol*	Azithromycin-like
$3A_1$	Mutator	4-aminopyridine*	Other
		Josamycin*	Azithromycin-like
		Phenethicillin	Ampicillin-like
		Phenylarsine oxide*	Other
		Puromycin*	Streptomycin-like
$3A_2$	Mutator	Chlorpromazine*	Ampicillin-like

		Josamycin	Azithromycin-like
		Puromycin	Streptomycin-like
		Thiamphenicol	Azithromycin-like
5A	Mutator	Josamycin	Azithromycin-like
		Minocycline	Streptomycin-like
		Phenethicillin	Ampicillin-like
		Puromycin	Streptomycin-like
		Spectinomycin	Streptomycin-like
		Thiamphenicol	Azithromycin-like

Table S11: MIC (measured as IC_{90} in µg/ml, methods) of the wild-type and mutator ancestral strains on the five antibiotics.

Ancestral strain	Ampicillin	Azithromycin	Nalidixic acid	Streptomycin	Trimethoprim
Wild-type	8	25.6	32	16	409.6
Mutator	8	51.2	128	32	819.2

Figure S8:

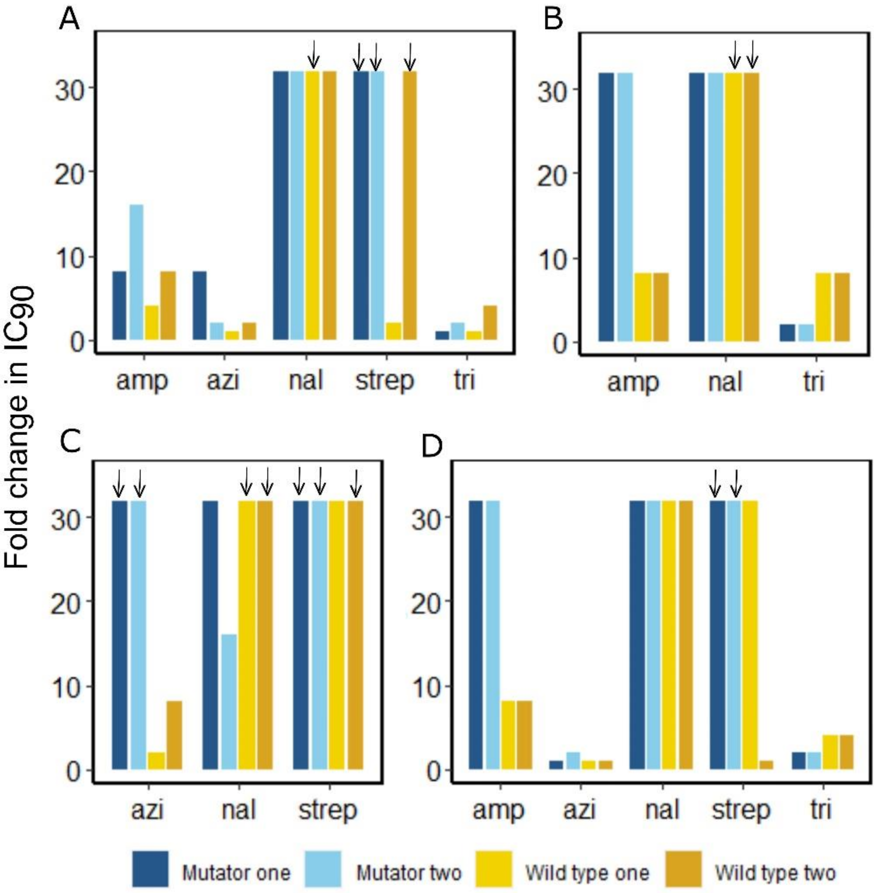

Figure S8: Fold-change in MIC (measured as IC_{90} in µg/ml, methods) for 32 representative clones on the antibiotics experienced during experimental evolution. A. Fold-changes in MIC for the two wild-type and two mutator clones evolved in five different single antibiotic environments. **B.** Fold-changes in MIC for the two wild-type and two mutator clones evolved in the $3A_1$ environment that contained ampicillin, nalidixic acid and trimethoprim. **C.** Fold-changes in MIC for the two wild-type and two mutator clones evolved in the $3A_2$ environment that contained azithromycin, nalidixic acid and streptomycin. **D.** Fold-changes in MIC for the two wild-type and two mutator clones evolved in the 5A environment, which contained all the five antibiotics. For every clone, the fold-change in MIC is shown relative to the MIC of the corresponding ancestral strain. Vertical downward-facing arrows '↓' indicate changes greater than 32-fold compared to the wild-type ancestor. Source data are provided as a Source Data file.

References -

1 Karve, S. & Wagner, A. Multiple novel traits originate non-adaptively in bacteria evolving on single antibiotics. *Molecular Biology and Evolution* (In review).

2 Sprouffske, K., Aguílar-Rodríguez, J., Sniegowski, P. & Wagner, A. High mutation rates limit evolutionary adaptation in Escherichia coli. *PLoS genetics* **14**, e1007324 (2018).

3 Ruiz, C., Levy, S. B. J. A. a. & chemotherapy. Many chromosomal genes modulate MarA-mediated multidrug resistance in Escherichia coli. **54**, 2125-2134 (2010).

4 Deveson Lucas, D. *et al.* Emergence of high-level colistin resistance in an Acinetobacter baumannii clinical isolate mediated by inactivation of the global regulator H-NS. **62**, e02442-02417 (2018).

5 Hugonnet, J.-E. *et al.* Factors essential for L, D-transpeptidase-mediated peptidoglycan cross-linking and β-lactam resistance in Escherichia coli. **5**, e19469 (2016).

6 Mainardi, J.-L., Villet, R., Bugg, T. D., Mayer, C. & Arthur, M. J. F. m. r. Evolution of peptidoglycan biosynthesis under the selective pressure of antibiotics in Gram-positive bacteria. **32**, 386-408 (2008).

7 Perron, K. *et al.* CzcR-CzcS, a two-component system involved in heavy metal and carbapenem resistance in Pseudomonas aeruginosa. *Journal of biological chemistry* **279**, 8761-8768 (2004).

8 Novak, R., Henriques, B., Charpentier, E., Normark, S. & Tuomanen, E. Emergence of vancomycin tolerance in Streptococcus pneumoniae. *Nature* **399**, 590-593 (1999).

9 Yamada, J. *et al.* Impact of the RNA chaperone Hfq on multidrug resistance in Escherichia coli. **65**, 853-858 (2010).

10 May, T., Ito, A. & Okabe, S. Induction of multidrug resistance mechanism in Escherichia coli biofilms by interplay between tetracycline and ampicillin resistance genes. *Antimicrobial agents and chemotherapy* **53**, 4628-4639 (2009).

11 Tanabe, H. *et al.* Growth phase-dependent transcription of emrKY, a homolog of multidrug efflux emrAB genes of Escherichia coil, is induced by tetracycline. **43**, 257-263 (1997).

12 Bennett, A. F., Lenski, R. E. & Mittler, J. E. Evolutionary adaptation to temperature. I. Fitness responses of Escherichia coli to changes in its thermal environment. *Evolution* **46**, 16-30 (1992).

13 Foster, P. L., Lee, H., Popodi, E., Townes, J. P. & Tang, H. Determinants of spontaneous mutation in the bacterium Escherichia coli as revealed by whole-genome sequencing. *Proceedings of the National Academy of Sciences* **112**, E5990-E5999 (2015).
