# Supplementary material for: Environmental complexity is more important than mutation in driving the evolution of latent novel traits in E. coli
Source: Nat Commun. 2022 Oct 6;13:5904. doi: 10.1038/s41467-022-33634-w (PMC9537139; doi:10.1038/s41467-022-33634-w)
Supplement: Supplementary file 3 — Reporting Summary [file 41467_2022_33634_MOESM3_ESM.pdf]

nature portfolio

Corresponding author(s): Shraddha Karve and Andreas Wagner

Last updated by author(s): Sep 16, 2022

Reporting Summary

Nature Portfolio wishes to improve the reproducibility of the work that we publish. This form provides structure for consistency and transparency in reporting. For further information on Nature Portfolio policies, see our Editorial Policies and the Editorial Policy Checklist.

Statistics

For all statistical analyses, confirm that the following items are present in the figure legend, table legend, main text, or Methods section.

n/a	Confirmed	
☐	☒	The exact sample size (*n*) for each experimental group/condition, given as a discrete number and unit of measurement
☐	☒	A statement on whether measurements were taken from distinct samples or whether the same sample was measured repeatedly
☐	☒	The statistical test(s) used AND whether they are one- or two-sided<br>*Only common tests should be described solely by name; describe more complex techniques in the Methods section.*
☐	☒	A description of all covariates tested
☐	☒	A description of any assumptions or corrections, such as tests of normality and adjustment for multiple comparisons
☐	☒	A full description of the statistical parameters including central tendency (e.g. means) or other basic estimates (e.g. regression coefficient) AND variation (e.g. standard deviation) or associated estimates of uncertainty (e.g. confidence intervals)
☐	☒	For null hypothesis testing, the test statistic (e.g. F, t, r) with confidence intervals, effect sizes, degrees of freedom and P value noted<br>*Give P values as exact values whenever suitable.*
☒	☐	For Bayesian analysis, information on the choice of priors and Markov chain Monte Carlo settings
☐	☒	For hierarchical and complex designs, identification of the appropriate level for tests and full reporting of outcomes
☒	☐	Estimates of effect sizes (e.g. Cohen's *d*, Pearson's *r*), indicating how they were calculated

Our web collection on statistics for biologists contains articles on many of the points above.

Software and code

Policy information about availability of computer code

Data collection	Tecan i-control (version 3.14)
Data analysis	R (version 3.5.2) was used for general statistical analysis. GrowthRates was used to determine the growth rates of the populations and the isolated clones. Integrative Genomics Viewer (version 2.9.2, Broad Institute, CA, US) was used for the visual inspection of assembled whole genome sequences and Breseq (version 0.35) was used for identifying the genetic variants.

For manuscripts utilizing custom algorithms or software that are central to the research but not yet described in published literature, software must be made available to editors and reviewers. We strongly encourage code deposition in a community repository (e.g. GitHub). See the Nature Portfolio guidelines for submitting code & software for further information.

Data

Policy information about availability of data

All manuscripts must include a data availability statement. This statement should provide the following information, where applicable:

- Accession codes, unique identifiers, or web links for publicly available datasets
- A description of any restrictions on data availability
- For clinical datasets or third party data, please ensure that the statement adheres to our policy

All data are available in the manuscript, the supplementary materials and source-data file. Whole genome sequencing data of the bacterial isolates is available from NCBI with Bioproject number PRJNA882999 (http://www.ncbi.nlm.nih.gov/bioproject/882999)

March 2021

Field-specific reporting

Please select the one below that is the best fit for your research. If you are not sure, read the appropriate sections before making your selection.

☐ Life sciences ☐ Behavioural & social sciences ☒ Ecological, evolutionary & environmental sciences

For a reference copy of the document with all sections, see nature.com/documents/nr-reporting-summary-flat.pdf

Ecological, evolutionary & environmental sciences study design

All studies must disclose on these points even when the disclosure is negative.

Study description	Experimental evolution, Biolog phenotypic assays and whole genome sequence analysis of Escherichia coli were used to assess the importance of environment and mutational supply in the evolution of novel traits without immediate benefits.
Research sample	Experimentally evolved bacteria of the strain MG 1655 of Escherichia coli. It is lab strain with two known varieties (wild type and mutator) which are handy to vary the mutation rates as our study demanded. We did not manipulate the strains in any way before starting the experimental evolution.
Sampling strategy	Sixteen independent experimental evolutions performed with eight replicate populations of Escherichia coli each. Two variants of the same bacterial strains with high and low mutation rates were evolved in eight different environments containing single or multiple antibiotics. The choice of sixteen independent experimental evolutions was dictated by logistic factors such as amount of time needed to setup each experiment at daily transfer.
Data collection	Evolved populations were analyzed at the end of the experimental evolution. Data was collected by Shraddha using Tecan i-control software (version 3.14) in Tecan Infinite PRO200 plate reader.
Timing and spatial scale	Evolved bacterial populations were sampled at the beginning (ancestor) and at the end of experimental evolution. Growth during the experimental evolution was noted at every 24 h interval. Experimental evolution lasted for ~147 days. The growth data in Biolog plates was collected after the experimental evolution over the duration of two days for representative clones from every environment*mutation rate combination. The measurements started in May 2018 and continued till August 2018 as per the availability of the plate reader.
Data exclusions	NA
Reproducibility	We used two evolved clones from every experimental evolution treatment for the Biolog phenotypic analysis and whole genome sequencing. We marked an evolution of novel trait in only those cases where none of the two ancestral clones showed growth while both the evolved clones could grow.
Randomization	As experimental evolution takes place in the precisely controlled incubator environment, randomization is not needed.
Blinding	We assayed all the ancestral and evolved clones for the evolution of novel traits and their genomic basis with a pre-decided criteria. Blinding was not applicable to our study as the properties we were assessing were only apparent after the measurement was made. In other words, the experimenter is blind to the results anyway (till the data analysis is done).

Did the study involve field work? ☐ Yes ☒ No

Reporting for specific materials, systems and methods

We require information from authors about some types of materials, experimental systems and methods used in many studies. Here, indicate whether each material, system or method listed is relevant to your study. If you are not sure if a list item applies to your research, read the appropriate section before selecting a response.

Materials & experimental systems

n/a	Involved in the study	
☒	☐	Antibodies
☒	☐	Eukaryotic cell lines
☒	☐	Palaeontology and archaeology
☒	☐	Animals and other organisms
☒	☐	Human research participants
☒	☐	Clinical data
☒	☐	Dual use research of concern

Methods

n/a	Involved in the study	
☒	☐	ChIP-seq
☒	☐	Flow cytometry
☒	☐	MRI-based neuroimaging
